# 

**Published:** 1892-06

**Authors:** 


CONTENTS—JUNE, 1892.—VOL. VII, No. 12.

ORIGINAL COMMUNICATIONS—

Notes on the Method of Preserving Bodies for Dissection at Medical Department, University of Texas, Galveston. Prof. Wm. Keiller, M. D., Professor of Anatomy . . . . . . . . . . . . . . . . . . . 425

Tubercular Peritonitis. T. J. Crofford, M. D . . . . 430

Purulent Pleurisy. W. W. Wallace, M. D . . . . . . 434

The Code of Ethics. E. J. Doering, M. D., Chicago . 437

Supra-pubic Cystotomy. L. A. Grizzard, M. D . . . 439

SELECTIONS—

Christian Science(?) . . . . . . . . . . . . . . . . . 440

Deaths from Chloroform . . . . . . . . . . . . . . . 442

Selected Foimulæ . . . . . . . . . . . . . . . . . . 443

EDITORIAL—

The Substitutor . . . . . . . . . . . . . . . . . . . 445

Libel Suit in England . . . . . . . . . . . . . . . . 446

Castration of Criminals . . . . . . . . . . . . . . . 448

MEDICAL NEWS AND MISCELLANY—

Numerous Items of Interest . . . . . . . . . . . . 448

In Memory of Dr. T. G. Richardson . . . . . . . . 451

PUBLISHERS' NOTES . . . . . . . . . . . . . . . . . 455

